# Hormonal cross-talk in plant development and stress responses

**DOI:** 10.3389/fpls.2013.00529

**Published:** 2013-12-24

**Authors:** Sergi Munné-Bosch, Maren Müller

**Affiliations:** Departament de Biologia Vegetal, Facultat de Biologia, Universitat de BarcelonaBarcelona, Spain

**Keywords:** hormonal balance, hormone interaction, hormone interplay, plant hormones, phytohormones

In contrast to animals, plants can continuously cease and resume growth. This flexibility in their architecture and growth patterns is partly achieved by the action of plant hormones. Plant hormones are structurally diverse compounds that act usually at nanomolar concentrations and include five groups of the so-called “classic” hormones, namely auxins, cytokinins, gibberellins, abscisic acid, and ethylene. Jasmonates, salicylates, strigolactones, brassinosteroids, polyamines, and some peptides were recognized as new families of plant hormones. Hormones build a signaling network and mutually regulate several signaling and metabolic systems, which are essential both for plant development and plant responses to biotic and abiotic stresses. Although earlier work greatly advanced our knowledge of how hormones affect plant growth and development and stress responses focusing on a single compound, it is now evident that physiological processes are regulated in a complex way by the cross-talk of several hormones. In this Research Topic, we aim at collecting a comprehensive set of original research and review papers focused on hormonal crosstalk in plants.

The goal of this Research Topic is to bring together recent work of experts studying hormonal crosstalk in plant development and stress response. Understanding how hormones and genes interact to coordinate plant growth is a major challenge in developmental biology. The activities of auxin, ethylene, and cytokinin depend on the cellular context and exhibit either synergistic or antagonistic interactions. Liu et al. (2013) use experimentation and network construction to elucidate the role of the interaction of the POLARIS peptide (PLS) and the auxin efflux carrier PIN proteins in the crosstalk of three hormones (auxin, ethylene, and cytokinin) in *Arabidopsis* root development. Naidoo et al. (2013) elegantly describe the transcriptional response of PR genes (*EgrPR2, EgrPR3, EgrPR4, EgrPR5, and EgrLOX*) identified in *Eucalyptus grandis* in response to SA and methyl jasmonate (MeJA) treatment. Blanco-Ulate et al. (2013) analyzed a transcriptome study of tomato fruit infected with *Botrytis cinerea* in order to profile the expression of genes for the biosynthesis, modification and signal transduction of ethylene, salicylic acid, jasmonic acid, and abscisic acid, hormones that may be not only involved in ripening, but also in fruit interactions with pathogens. The changes in relative expression of key genes during infection and assays of susceptibility of fruit with impaired synthesis or perception of these hormones were used to formulate hypotheses regarding the involvement of these regulators in the outcome of the tomato fruit–*B. cinerea* interaction.

A series of reviews also add to the current knowledge of hormonal cross-talk in the regulation of plant development and stress responses. Arc et al. (2013) review our current knowledge of ABA crosstalk with ethylene and NO, both volatile compounds that have been shown to counteract ABA action in seeds and to improve dormancy release and germination. McAtee et al. (2013) review current evidence on the topic and elegantly describe the hormonal cross-talk in the developing seed and its surrounding fruit tissue during fruit development. Denancé et al. (2013) address novel insights on the regulatory roles of the ABA, SA, and auxin in plant resistance to pathogens and describe the complex interactions among their signal transduction pathways. The strategies developed by pathogens to evade hormone-mediated defensive responses are also reviewed. Based on these data it is also discussed how hormone signaling could be manipulated to improve the resistance of crops to pathogens. From another perspective, Daszkowska-Golec and Szarejko (2013) review recent findings on phytohormone crosstalk, including changes in signaling pathways and gene expression that impact on modulating stress response through the closing or opening of stomata. da Costa et al. (2013) review current evidence indicating a clear hormonal cross-talk in the regulation of adventitious rooting. Cheng et al. (2013) review current evidence on the recently discovered phytohormone class, strigolactones and their cross-talk with other plant hormones—such as auxin, cytokinin, abscisic acid (ABA), ethylene (ET), and gibberellins (GA)—during different physiological processes. Finally, O'Brian and Benkkova discuss the complex hormonal cross-talk in plant responses to environmental stress, with a focus on cytokinins and other hormones, such as abscisic acid, jasmonates, salicylates, ethylene, and auxin. Of particular interest is the discussion of the impact of this research in the biotechnological industry.

In conclusion, taken together these original and review articles reflect the explosion of interest and considerable progress that has recently been made in the dynamic field of plant biology, with a particular focus on better understanding hormonal cross-talk in plant development and stress responses. It will be intriguing to see how future work on hormonal cross-talk in plants will continue. We hope that the articles that have been compiled will provide new insights into this topic and shed new light concerning the complex but exciting phenomenon of hormonal cross-talk in plant development and stress responses.
